# Anti-viral RNA silencing: do we look like plants ?

**DOI:** 10.1186/1742-4690-3-3

**Published:** 2006-01-12

**Authors:** Anne Saumet, Charles-Henri Lecellier

**Affiliations:** 1 CNRS UPR2357, Institut de Biologie Moléculaire des Plantes, 12, rue du Général Zimmer, 67084 STRASBOURG Cedex, France

## Abstract

The anti-viral function of RNA silencing was first discovered in plants as a natural manifestation of the artificial 'co-suppression', which refers to the extinction of endogenous gene induced by homologous transgene. Because silencing components are conserved among most, if not all, eukaryotes, the question rapidly arose as to determine whether this process fulfils anti-viral functions in animals, such as insects and mammals. It appears that, whereas the anti-viral process seems to be similarly conserved from plants to insects, even in worms, RNA silencing does influence the replication of mammalian viruses but in a particular mode: micro(mi)RNAs, endogenous small RNAs naturally implicated in translational control, rather than virus-derived small interfering (si)RNAs like in other organisms, are involved. In fact, these recent studies even suggest that RNA silencing may be beneficial for viral replication. Accordingly, several large DNA mammalian viruses have been shown to encode their own miRNAs. Here, we summarize the seminal studies that have implicated RNA silencing in viral infection and compare the different eukaryotic responses.

## Introduction

RNA silencing is often considered as a potent nucleic acid-based immune system. In fact, invading nucleic acids can be recognised by some cells as undesirable, by a mechanism that is not yet totally unravelled, and are silenced by a process based on 21–25 nt long small RNAs. A now classical example of this phenomenon was provided more than ten years ago by experiences performed on transgenic petunias [1,2]. Initially, these plants had been engineered to produce more flower pigments and the strategy was to introduce extra copies of the gene encoding the chalcone synthase (CHS). However, a non-negligible proportion of the transformants did not show flowers with the expected purple colour but, rather, the flowers were completely white, with no pigment. Because both the transgene and the endogenous CHS mRNAs were affected in a nucleotide-sequence homology manner, this phenomenon was coined 'co-supression'. Later on, similar gene silencing phenomena were reported in other eukaryotes, including fungi [3] and worms [4], and the molecular basis of RNA silencing began to be clarified (for a recent review [5]). The initiation of silencing necessitates the synthesis of double-stranded RNAs (dsRNAs, produced by various mechanisms e.g. viral replication) that is further cleaved by an RNAse type III enzyme, called Dicer, into 21–25 nt long small RNAs. These small RNAs are the *trans*-acting determinants of RNA silencing and a core feature detected each time silencing is triggered. They direct a multi-component complex, the RNA-induced silencing complex (RISC), on a targeted mRNA harbouring sequence-homology. RISC invariably contains some Argonaute (Ago) family member proteins, such as Ago2 in human [6], that provide endonucleolytic activity to the complex. The first discovered natural function of RNA silencing was anti-viral response, again in plants [7], wherein replication of RNA and DNA viruses is associated with the accumulation of virus-derived small RNAs. These small RNAs are thought to trigger the cleavage of viral messengers and, hence, to limit viral infection. Because the essential silencing components, notably Dicer and Ago proteins, are found in most organisms, the idea that RNA silencing functions, particularly in anti-viral defence, are also conserved, rapidly emerged. Here, we review the decisive studies that implicated RNA silencing in the replication of viruses, from plant to human, and compare the underlying mechanisms.

### Anti-viral silencing in plants

#### Virus-derived siRNAs

Several observations from plant virologists converged to the idea that RNA silencing was an efficient anti-viral system. The first evidence probably came with the finding that plant viruses trigger the silencing of endogenous mRNAs sharing sequence-homology. For instance, the phytoene desaturase (PDS) mRNA was easily silenced upon replication of the Tobacco mosaic virus (TMV) harbouring a stretch of PDS [8]. This led to the development of an outstanding reverse genetic tool, now widely used in plant biology, known as Virus-induced gene silencing (VIGS). The phenomenon of "recovery" further demonstrated that plant viruses are targeted by RNA silencing: when transgenic plants, expressing the coat protein (CP) of Tobacco etch virus (TEV), were infected with TEV, symptoms clearly appeared in the inoculated leaves but progressively disappeared in the new growth, which became in turn resistant to super-infection with TEV [9]. This resistance was associated with a complete degradation of the mRNAs of both TEV and CP transgene. The recovery was thereafter shown to be naturally elicited by some plant viruses infecting non transgenic wild type plants [10,11]. RNA silencing also helped explaining the phenomenon of "cross-protection" whereby attenuated strains of a given virus are used to immunise plants against aggressive strains of the same virus [12]. This is exemplified with plants infected with a recombinant Potatoe Virus X (PVX) carrying a GFP insert that become resistant to Tobacco mosaic virus (TMV) infection carrying the same insert [13]. But the definitive proof that plant viruses triggered RNA silencing was provided by the demonstration that virus-derived siRNAs accumulate to high levels in plants during the course of infection [14]. In fact, dsRNA replication intermediates of RNA viruses, the vast majority of plant viruses, and/or high secondary structures of single stranded RNAs (ssRNAs, notably for the few DNA plant viruses) are thought to constitute the substrate of at least one of the plant Dicer homologues (4 in the plant model *Arabidopsis thaliana*) [15]. The Dicer like 2 (DCL-2) was shown to produce the siRNAs derived from the turnip crinkle virus (TCV), but not those from the cucumber mosaic virus strain Y (CMV-Y) or the turnip mosaic virus (TMV). Additionally, Xie *et al*., have shown that the replications of CMV-Y and TMV were not affected in plants impaired in DCL-1 and DCL-3 functions, likely suggesting that DCL-4 functions as a component of the anti-TMV and anti-CMV silencing [15]. At that point, we can already catch a glimpse at the complexity of the RNA silencing pathway in plants, wherein each DCL is thought to be specialised in a particular pathway (although some redundancy are possible [16]) a situation that may not be encountered in worm or human, which harbour only one Dicer gene [17]. In fact, plant cells naturally produce numerous sub-classes of small RNAs, involved for instance in epigenetic modification and biogenesis of other small RNAs, that are not yet found in human cells [18,19].

#### Viral suppression of RNA silencing

An indirect proof that RNA silencing constitutes an efficient anti-viral system was also provided by the discovery of virus-encoded suppressors of silencing. The observation of an accentuation of symptoms induced by one virus by co-infection with a second and unrelated virus, a phenomenon called synergism, provided the first hint for virus-mediated silencing suppression [20]. The Potyvirus Y (PVY) dramatically enhances the replication of PVX when co-inoculated, suggesting that PVY encodes a suppressor of host defence [20]. Among the PVY proteins, the helper component proteinase (HcPro) was sufficient to recapitulate the molecular and symptomatic effects of PVY on PVX [21,22]. The demonstration that HcPro is a genuine suppressor of silencing came with the observation that HcPro specifically affected gene silencing directed against a GFP reporter gene [23]. Following these observations, it was shown that silencing suppression is a common property of most, if not all, plant viruses [24]. Interestingly, these proteins are extremely diverse in sequence and structure and are encoded by both DNA and RNA viruses [24]. This strongly suggests a vast diversity in their mode of action and, therefore, viral suppressors are thought to affect all steps of RNA silencing, in this manner being very useful to dissect molecular basis of RNA silencing [25,26]. Probably the most studied viral suppressor is the P19 protein of tombusvirus. Gel mobility shift assays showed that the P19 protein of Cymbidium Ringspot Virus (CymRSV) exclusively binds to 21 nt-long dsRNA with 2 nt-long 3' overhanging ends, a characteristic of authentic siRNAs, but not to long ssRNA, dsRNA or ss siRNAs [27]. Moreover, P19 of Tomato Bushy Stunt Virus (TBSV), closely related to CymRSV, co-immunoprecipitates with siRNAs in *planta *[26]. The crystal structures of p19 from TBSV and the Carnation Italian Ringspot Virus (CIRV), bound to a 21 nt siRNA, demonstrated that tombusviral P19 protein acts as a molecular caliper to specifically select siRNAs based on the length of the duplex region of the RNA, in a sequence-independent manner [28,29]. Therefore, P19 likely sequesters siRNAs and, thereby, prevents their incorporation into the RISC complex. Because siRNAs are ubiquitous effectors of silencing, we anticipated that P19 should exert its effect in a broad range of organisms and, accordingly, we demonstrated that P19 inhibits RNA silencing triggered by synthetic siRNAs in human Hela cell line [26].

#### Non-cell autonomous RNA silencing

The capacity of plant RNA silencing to be amplified and to propagate in the whole organism likely represent two additional layers that ensure its efficacy against viruses [30]. The plant genome encode several RNA-dependent RNA polymerase (RdRp), among those the RDR6, thought to recognize and to use as template undesirable transcripts such as transgene or viral mRNAs [31]. RDR6 synthesised a complementary strand from a ssRNA, resulting in the production of dsRNA, which is, in turn, processed by Dicer to generate more siRNAs. RDR6 activity also forms the basis of a silencing-related phenomenon, coined transitivity, which is responsible of an amplification in the siRNA production [32,33]. When silencing is elicited against a precise sequence stretch of a targeted RNA, it first generates 'primary' siRNAs perfectly complementary to this particular stretch. But 'secondary' siRNAs are also detectable, upstream or downstream the initial stretch, likely reflecting a combined action of one DCL and RDR6 [34,35]. The final result of transitivity is the production of more siRNAs that do not necessarily share sequence-homology with the initial target [33]. Transitivity is also implicated in the propagation of silencing and in its non-cell autonomous effects [35]. As described above, the activation of silencing first results in the production of siRNAs, at the single cell level. Rapidly after induction, silencing manifestations are also detectable around the zone of initiation, corresponding to a nearly constant number of 10–15 cells. This RDR6-independent short-range movement is thought to initiate an RDR6-dependent long distance propagation of silencing: the primary siRNAs diffuse outside this 10–15 cells border and mediate the production of secondary siRNAs through the action of RDR6 that use a sequence-homologous transcript as a template. These secondary siRNAs are then able to move in surrounding cells and to reiterate the production of siRNAs leading to a systemic propagation of silencing, in a relay-amplification manner [35,36]. The requirement of transitivity and silencing movement for viral defence is illustrated by the observations that plants compromised in RDR6 are hyper-susceptible to some viruses [37]. It is conceivable that the propagation of RNA silencing ensures the immunization of naive cells before the ingress of the virus. The existence of silencing suppressors, able to specifically inhibit silencing movement, is again consistent with the relevance of that phenomenon in anti-viral response [38].

From those studies, we may consider that the demonstration that a non-plant virus is restricted by RNA silencing requires three experimental observations: (i) presence of virus-derived siRNAs, illustrating the onset of RNA silencing, (ii) production of a virus-encoded silencing suppressor, as a mechanism to escape these virus-derived siRNAs and (iii) silencing movement in the infected host, which may be an indirect hint for the efficiency of anti-viral silencing.

### Anti-viral RNA silencing in invertebrates

#### Insect

Many arthropod species have been found to support artificially induced RNA silencing, among which fruit flies [39] and mosquitoes [40] but the first evidence for a contribution of silencing in anti-viral defence came in 2002, from decisive experiments performed in *Drosophila *S2 cells infected with the Flock House Virus (FHV), member of the *Nodaviridae *family [41]. Li *et al*., reported the accumulation of virus-derived siRNAs in FHV-infected S2 cells. The viral accumulation was further found to be enhanced in cells depleted for the AGO2 protein, a crucial component of the RISC complex, as mentioned above [41]. Determinedly, FHV encodes a silencing suppressor, namely B2, that is functional in both insects and plants, indicating that the steps and/or components of silencing that are targeted by B2 are shared by those two organisms [41]. Recent studies indeed showed that B2 binds dsRNA without regard to length and inhibits cleavage of dsRNA by Dicer *in vitro *[42,43]. Second, similar to plant, VIGS has also been documented in the silkmoth Bombyx mori wherein the transcription factor Broad-Complex (BR-C) was silenced by infection with a recombinant Sindbis alphavirus expressing a BR-C antisense RNA [44]. Although these experiments clearly demonstrate that insect cells are able to mount an anti-viral response based on the activation of the silencing pathway, it remains to be determined if this response is also efficient in the whole organism. An important issue is to determine whether non-cell autonomous silencing operates in insects, similarly to what is observed in plants. Although Lipardi *et al*., reported an RdRp activity in *Drosophila *embryonic extracts [45], no member of the RdRp gene family can be identified in the *Drosophila *genome. More important, using transgenes expressing dsRNA in adult fly, Roignant *et al*., have conclusively demonstrated that transitive RNA silencing does not occur in *Drosophila *and that it remains strictly confined within the cells where it as been elicited [46]. Thus, the question remains open as to know whether RNA silencing is an efficient component of the insect anti-viral response. Nonetheless, an indirect clue for natural RNA silencing directed against exogenous viruses in insect may be provided by the mechanism that has been elaborated by the *Drosophila *genome to domesticate endogenous and mobile genetic elements. Jensen *et al*., reported that transpositional activity of the I element, a transposon similar to mammalian LINE elements, can be repressed by prior introduction of transgenes expressing a small internal region of the I element [47]. This regulation presented features characteristic of the co-suppression initially observed in plants since, notably, it did not required any translatable sequence. Furthermore, Sarot *et al*., reported that the endogenous retrovirus *gypsy *is silenced in fly ovaries by the action of one argonaute protein and that ovary cells naturally accumulate *gypsy*-derived small RNAs [48]. RNA silencing directed against endogenous and invasive sequences appears therefore very similar to those directed against exogenous pathogens. However, the production of a silencing suppressor by an endogenous (retro)element has never been reported so far. Interestingly, this transposon taming is also found in plants in which silencing is clearly efficient against exogenous viruses [49]. Hence, the presence of a silencing-mediating transposon taming may represent another hint for the existence of anti-viral RNA silencing.

#### Nematodes

Transposable elements are also tamed in *Caenorhabditis elegans *by a mechanism related to RNA silencing. Sijen *et al*., detected dsRNAs and siRNAs derived from diverse regions of the Tc1 transposon and showed that a germline-expressed reporter gene, fused to a stretch of the Tc1 sequence, is silenced in a manner dependent on essential silencing components [50]. Cloning of endogenous small RNAs also yielded several siRNAs corresponding to Tc1 [51]. As mentioned above, these findings may be informative about the potential implication of RNA silencing in the worm anti-viral defence. One indirect evidence may come from the observation that, in contrast to *Drosophila*, RNA silencing moves in worm. In a shaping study wherein they demonstrated that dsRNA is the key elicitor of RNA silencing, Fire *et al*. also reported that injection of dsRNA into the body cavity or gonad of young adults produced gene-specific interference in somatic tissues of the injected animal [4]. The *C. elegans *genome contains 2 RdRp genes, termed *ego-1 *and *rrf-1*, mandatory for RNA silencing in germline and somatic tissues, respectively [33,52]. However, the obligate necessity of RdRp activity for RNA silencing in nematodes makes it hard to determine whether it is required for propagation, like in plant. Nonetheless, Alder *et al*., reported that mRNA targeted by RNA silencing functions as a template for 5' to 3' synthesis of new dsRNA [53]. This effect was non-cell autonomous since dsRNA targeted to a gene expressed in one cell type can lead to transitive RNAi-mediated silencing of a second gene expressed in a distinct cell type. To better understand the molecular basis of silencing movement in worm, two groups designed genetic screens and isolated defective mutants, called *sid *(systemic RNAi defective) [54] and *rsd *(RNAi-spreading defective) [55]. Both groups identified a particular gene, called *sid-1/rsd-8*, encoding a multispan transmembrane protein essential for systemic but not cell-autonomous RNAi [54,55]. Feinberg *et al*., further demonstrated that SID-1 facilitates the passive cellular uptake of preferentially long dsRNAs using *Drosophila *S2 cells [56]. Interestingly, SID-1 is found in human cells where it localizes to the cell membrane and enhances the passive transport of siRNAs, resulting in an increased efficacy of siRNA-mediated gene silencing [57].

In nematodes, the mechanism of transposon taming and the movement of RNA silencing together suggest that silencing is implicated in anti-viral defence. However, asking whether silencing is involved in worm anti-viral defence is complicated by the absence of worm-specific viral pathogens (although some plant viruses use nematodes as transmission vectors [58]). Nonetheless, the Ding and the Machaca groups recently reported that two non-natural viruses efficiently trigger anti-viral RNA silencing in *C. elegans *[42,59]. Wilkins *et al*., showed that the nematode N2 cells do support the replication of the mammalian Vesicular Stomatitis Virus (VSV) [59]. VSV replication is enhanced in silencing defective worm mutants, impaired in the RDE-4-RDE-1 complex, thought to recognize dsRNA and to target it for cleavage into siRNAs by Dicer. Conversely, VSV replication is inhibited in mutant nematodes impaired in the functions of RFF-3 and ERI-1, two negative regulators of RNA silencing. RRF-3, a member of the RdRP gene family in *C. elegans*, seems to inhibit RdRP-directed siRNA amplification, and worms with mutations in *rrf-3 *are more sensitive to RNA silencing induced by dsRNAs [60]. ERI-1, a member of the DEDDh nuclease family, preferentially cleaves siRNAs, which are in turn more stable and accumulate in *eri-1 *mutants, resulting in enhanced gene suppression [61]. Decisively, Wilkins *et al*., observed virus-specific 20–30 nt long small RNAs [59]. Likewise, Lu *et al*., showed complete replication of FHV in worm strains carrying integrated transgenes coding for full-length cDNA copies of FHV genomic RNAs [42]. The anti-FHV response required the RDE-1 activity and could be suppressed by the FHV-encoded B2 silencing suppressor [42]. The fact that *C. elegans *is able to respond to viral infection by generating virus-derived siRNAs may indicate that the complexity of the silencing pathways (e.g. 4 dicers in *Arabidopsis thaliana*, 2 in *Drosophila *and only one in nematode) is not a prerequisite for the existence of anti-viral RNA silencing.  This is particularly important when investigating the potential anti-viral role of silencing in mammals, which, like worms, encode only one Dicer. However, we cannot yet exclude that the complexity of the silencing pathway is ensured by the diversity of the Argonaute proteins found in worm [62].

### What about mammals?

We can now assume that anti-viral RNA silencing exists in plant, insect and nematode, even if the question as to know whether it is a natural and efficient anti-viral response in invertebrates remains opened. For that reason, several laboratories were prompted to investigate the potential contribution of RNA silencing in the replication of mammalian viruses.

#### Virus-encoded miRNAs but no virus-derived siRNAs

**Figure 1:**
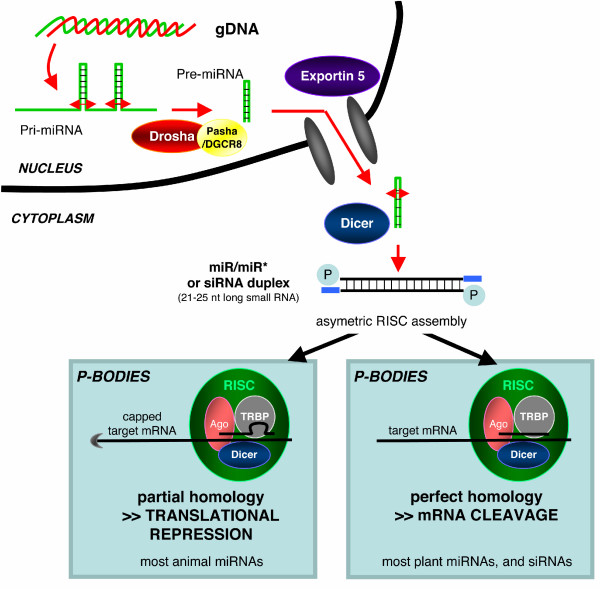
**miRNA biogenesis and action**. Long primary transcripts (pri-miRNAs) containing one or several miRNAs are transcribed by RNA polymerase II and cleaved by the Microprocessor Complex, containing at least Drosha (RNAase III endonuclease) and DGCR8/Pasha in human (a double-stranded RNA binding protein). This complex recognizes the double stranded RNA structure of the pri-miRNA and specifically cleaves at the base of the stem loop, hence releasing a 60- to 70-nucleotide precursor(pre)-miRNA. This pre-miRNA is then exported through the Exportin-5 pathway into the cytoplasm where it is further processed into a mature miR/miR* duplex by Dicer, a second RNase III endonuclease. The miR/miR* duplex is then loaded into a multi-component complex, the RNA-induced silencing complex (RISC), constituted of at least TRBP (TAR Binding Protein), Dicer, and one Argonaute (Ago2 in human). The miR serves as a guide for target recognition while the miR* passenger strand is cleaved by Ago2. In contrast to siRNAs (small interfering RNA) and plant miRNAs, which induced the cleavage of the targeted mRNA, most of animal miRNAs harbour an imperfect homology with their targets and, therefore, inhibit translation by a RISC-dependent mechanism that probably interferes with the mRNA cap recognition. This step occurs in cytoplasmic foci called P-bodies (for processing bodies), which contain untranslated mRNAs and can serve as specific sites for mRNA degradation.

The Tusch1 lab first attempted to clone virus-derived siRNAs from cells infected with various viruses [63]. They neither found virus-derived siRNAs nor endogenous small RNAs derived from transposable or repetitive elements, suggesting that, unlike in plant, insect and worm, mammalian transposable elements are not naturally tamed by a silencing-related mechanism. They rather found discrete species of small RNAs encoded by the Epstein-Barr Virus, very akin to endogenous host-encoding small RNAs found in eukaryotic cells and involved in the control of genome expression: the micro(mi)RNAs [63] (Figure 1). More than 300 miRNAs are now described in humans but their exact function still remains largely obscure (for review [64,65]). One reason may lie in the mode of action of animal miRNAs: in contrast to siRNAs, most animal miRNAs harbour an imperfect homology with their target and, therefore, miRNAs are thought to not affect RNA stability but rather inhibit translation by a RISC-dependent mechanism. This absence of perfect homology considerably limits the identification of miRNA cellular targets. It has recently been shown that miRNAs probably interfere with the mRNA cap recognition [66,67]. However, in addition to the previously described exception of miR-196 and its target HoxB8 [68], recent report also suggest that miRNAs may broadly affect RNA stability, despite imperfect sequence homology [69,70]. Basically, the miRNA genes are transcribed by RNA polymerase II into primary(pri)-miRNA, which are cleaved by a nuclear RNAse III, coined Drosha, into precursor(pre)-miRNA (Figure 1) [71-74]. This pre-miRNA is exported from the nucleus through the Exportin-5 pathway into the cytoplasm where it is further processed into a miR/miR* duplex by Dicer [75]. The duplex is then loaded into the RISC complex and the miR serves as a guide for target recognition whereas the passenger miR* is cleaved by Ago2 [76,77]. Although miRNAs encoded by other viruses, in particular HIV, have been predicted [78-80], virus-encoded miRNAs seem to be defining for large DNA viruses, which replicate in the nucleus, such as Herpesviruses (Kaposi sarcoma herpesvirus KSHV, mouse gammaherpesvirus MGHV, human cytomegalovirus HCMV, for instance), Polyomaviruses (Simian Virus 40 SV40, Simian Agent 12 SA12) and Adenovirus (for review [81]). Like their cellular counterparts, those viral miRNAs are transcribed by RNA polymerase II and are thought to follow the same biogenesis (with the notable exception of MGHV miRNAs which are predicted to be pol Ill-transcribed [82]). The exact function(s) of the viral miRNAs are not yet known except in the case of the SV40 miRNA which mediates the degradation of the perfectly complementary transcript encoding large T antigen [83]. This may help the virus to escape the immune response, notably the cytotoxic T cells, by limiting the production of viral antigens. Some herpesvirus-encoded miRNAs are also perfectly complementary to cognate viral transcripts suggesting that they could mediate RNA cleavage and regulate the translation of viral proteins [63]. Moreover, virus-encoded miRNAs may regulate the translation of cellular messengers to create favourable conditions for viral replication but this remains to be firmly established.

#### Contribution of cellular miRNAs but still no virus-derived siRNAs

By our side, we also started working on the contribution of RNA silencing in mammalian anti-viral response, using the prototypic foamy retrovirus, the Primate Foamy Virus type 1 (PFV-1), as a model [84]. This complex retrovirus, akin to HIV or HTLV-I, was chosen because (i) siRNAs derived from LTRs of endogenous retroviruses have been described in various eukaryotes [85,86], (ii) PFV-1 was shown to retrotranspose in the genome of the infected cell, a feature that is so far unique among retroviruses [87] and (iii) the latency induced by PFV-1 is closely similar to the 'recovery' observed with some plant viruses [88-90]. First, we used the TBSV P19 protein to inhibit RNA silencing in mammalian cells and showed that, upon P19 expression, PFV-1 replication was dramatically increased, suggesting that a silencing-related pathway limits viral infection [84]. We then tried and failed to isolate virus-derived siRNAs during acute or latent infections and in various cell lines. However, during the course of this study, we observed that a cellular miRNA, namely the miR-32, efficiently inhibits the replication of PFV-1 by hybridizing with the 3'UTR of viral mRNAs.

The anti-viral effect of miR-32 was not linked to a potential implication of its unknown cellular target because a mutant carrying point mutations, that disrupt the hybridization of the cellular miRNA with the viral mRNA, accumulated to higher level than the wild type virus [84]. This observation suggested that cellular miRNAs, by recognizing foreign and, in particular viral, mRNAs, have the potential to limit viral replication. Because exogenous viruses are not transmitted through the germen of infected hosts, it is unlikely that the mammalian genomes have evolved to specifically encode miRNAs whose sole function would be to regulate translation of exogenous and viral transcripts. We rather propose that the functional interactions between cellular miRNAs and viral mRNAs are governed by fortuitous micro-homology. The fact that the core activity of a miRNA resides in its 7–8 first nucleotides, known as the "miRNA seed" [91,92], extends the chances of fortuitous recognition of exogenous transcripts and implies that this miRNA-based anti-viral silencing may fell beyond the case of PFV-1. In fact, targets for cellular miRNAs have been predicted in several and unrelated viral genomes using miRNA target prediction algorithms [84,93].

Cellular miRNAs are implicated in fundamental biological processes, such as cellular differentiation for instance, therefore, each cell type is thought to harbour a particular miRNA repertoire [64]. In that case, miRNAs may participate in cellular permissivity because a virus would replicate in cell types, where the 'anti-viral' miRNAs are less or not produced. The findings by Stones *et al*., that gene therapy viral vectors containing a miRNA target exhibit a tissue-specific expression according to miRNA expression levels support this proposal [94]. Besides, we demonstrated that the anti-viral functions of cellular miRNAs are not necessarily linked to their cellular functions [84], raising the possibility that miRNAs may be expressed differently in a specific tissue (where they do not play a crucial role) in different individuals. Hence, cellular miRNAs may also participate in the individual susceptibility to viral infection.

Viral genomes have alas the capacity to rapidly and non-randomly evolved, notably to counteract therapeutic strategies and to settle in new cellular contexts. Nonetheless, it appears that PFV-1 have conserved the viral target of miR-32, suggesting that PFV-1 may hijack the miR-32, for instance, to decrease viral protein expression during the latent stage of infection [88-90]. In line with this, Switzer *et al*., have shown that simian foamy viruses might have co-speciated with their Old World primate hosts for at least 30 million years [95]. A recent study by the Sarnow group provided an explicit proof for a positive role of a cellular miRNA in viral replication [96]. They demonstrated that Hepatitis C Virus (HCV) replication requires the expression of the miR-122, an abundant liver-specific miRNA. In fact, a genetic interaction between miR-122 and the 5' noncoding region of the HCV genome was highlighted by mutational analyses of the predicted microRNA binding site and ectopic expression of miR-122 molecules containing compensatory mutations. Curiously, miR-122 did not detectably affect mRNA translation nor RNA stability [96]. The authors rather proposed that miR-122 is involved in the folding of viral RNAs and/or redirects viral RNAs to particular sites of replication [96]. Another hint for the positive requirement of cellular miRNAs in viral replication may indeed be illustrated by miRNAs encoded by large DNA viruses. In fact, these viruses are known to efficiently usurp cellular pathways and to integrate cellular genes inside their genomes, even to modify them for their own advantage (e.g. cytokines, receptors of cytokines) [97]. Thus, cellular miRNAs may constitute the source and the origin of viral miRNAs.

#### Silencing suppression by mammalian viruses : more miRNAs?

To escape this miRNA-based 'innate' form of immunity, we additionally showed that PFV-1 encodes a suppressor of silencing, Tas, that have the capacity to inhibit miR-32 action [84]. Tas exerts its effect not only in mammalian cells but also in plants, where it inhibits RNA silencing triggered by an inverted repeat against an endogenous gene. Tas is the foamy viral transactivator that activates the 5'LTR and an internal promoter located at the 3' end of the *env *gene [98,99]. In contrast to HIV Tat or HTLV-I Tax, Tas directly binds DNA, although no precise consensus sequence can be characterized [98-101]. Interestingly, those two functions, i.e. transactivation and silencing suppression, are shared with the AC2 protein of the plant geminivirus [24], likely reflecting a convergent evolution in viral replication strategy. Several suppressors of silencing encoded by mammalian viruses are now identified, either as protein or RNA form. For instance, Adenovirus encodes the small VA1 RNA, analogous to a miRNA precursor, that titers the miRNA biogenesis pathway [102]. The Influenzae NS1 binds siRNAs and impedes silencing, at least in plant, but its action in mammalian cells remains to be verified [103-105]. More recently, HIV-1 Tat has been shown to inhibit Dicer activity, independently of its transcriptional function [106]. Several of those suppressors (Tas, NS1, VA1) have been shown to non-specifically affect the action of cellular miRNAs [84,102,107]. Because miRNAs are thought to be essential for the cellular biology, the perturbation of their action by these virulent factors may participate in the development of the cytopathic effects associated with the infection.

An alternative strategy to escape cellular miRNAs could be to introduce synonymous mutations in the viral genome that would disrupt the cellular miRNA/viral target hybrid. This hypothesis may be suitably applied to high mutation rate viruses. In fact, this particular type of RNA silencing evasion has already been described for HIV and HCV when artificially targeted by synthetic siRNAs [108-110]. As a consequence, the synthetic siRNAs can influence the emergence of the viral quasi-species, as reported in plants, wherein virus-derived siRNAs influence the emergence of defective interfering RNA viruses [111]. This scenario may also be envisaged for cellular miRNAs.

## Conclusion

Recent evidences support a role for RNA silencing in the replication of mammalian viruses but its consequences remain to be clarified as to know whether it is positively required for replication or if, conversely, it constitutes a crucial host defence system. To date, only miRNA molecules, either encoded by the host or by the virus itself, have been implicated, with the notable exception of one discret HIV-derived siRNA duplex produced during the course of infection and able to cleave Env mRNA [106]. The mode of action of miRNAs, that requires precise targeted sequences, may argue against the existence of virus-derived siRNAs, like it is encountered in plant, insect or nematode. For instance, in the case of SV40, it would be hard to reconcile the regulation of large T Antigen by a specific viral miRNA in the presence of several siRNAs, derived from the whole viral genome, and able to indiscriminately cleave viral messengers. Of course, we cannot exclude the possibility that these two small RNA species (i.e. viral miRNA and virus-derived siRNAs) are not produced during the same steps of viral replication. Alternatively, virus-derived siRNAs could be produced in specialized cells, which have not yet been characterised, and then propagate in the rest of the organism, likely through the blood vessels. This hypothesis is supported by (i) several studies that clearly demonstrate the effective inhibition of the replication of several mammalian viruses with artificially delivered siRNAs [112] and (ii) the existence of silencing propagation, via SID-1, in mammalian cell culture [57]. Moreover, chemically synthesised siRNA have been shown to naturally enter epithelial cells of the mouse jejunum after intravenous administration, although cholesterol conjugation drastically increases this ability [113].

Finally, long dsRNAs typically used to elicit RNA silencing in other organisms potently activate a specific mammalian cell defence mechanism, the Interferon (IFN) Response [114] (for review on IFN [115]). This non-specific response was first reported in 1957 by Isaacs and Lindenmann who showed that influenza virus-infected chick cells secreted a factor that could, on its own, activate an antiviral state when brought into contact of naive cells [116,117]. In contrast to RNA silencing, this antiviral state is broadly effective, as it could target both sequence homologous and heterologous viruses. IFN response often leads to cell death mainly due to a global shut-off in protein expression (via the protein kinase R and the phosphorylation of the α subunit of the protein synthesis initiation factor 2) and a non-specific RNA degradation (through the action of the 2',5'-oligoadenylate synthetase and the RNaseL). Therefore, IFN might be considered as a programmed suicide developed by infected cells to protect naive cells from becoming infected. Interestingly, RNA silencing and IFN response seem to be partially overlapping because, for instance, (i) the double-stranded RNA-specific adenosine deaminase ADAR edits miRNA precursor and is also an effector of the IFN response [118], (ii) some viral products efficiently inhibit both RNA silencing and IFN response by targeting their common elicitor, dsRNA [103], and (iii) the TAR Binding Protein (TRBP), which is a negative regulator of PKR, is an essential component of the RISC [119-121]. In fact, it is currently thought that RNA silencing and IFN pathway even antagonize [122-124]. Hence, the differences in anti-viral RNA silencing observed between plant, insect and nematode in one hand and mammals in the other may lie in the existence of this mammalian-specific IFN system. This aspect may be appropriately studied in the marine shrimp wherein dsRNA induces both sequence-specific anti-viral silencing, similar to plant or insect, and non-specific immunity [125].

## Competing interests

The author(s) declare that they have no competing interests.

## Authors' contributions

AS and CHL participated to the conception, design and writing of the article.
